# Supplementary material for: Trends and associated factors for Covid-19 hospitalisation and fatality risk in 2.3 million adults in England
Source: Nat Commun. 2022 Apr 29;13:2356. doi: 10.1038/s41467-022-29880-7 (PMC9054846; doi:10.1038/s41467-022-29880-7)
Supplement: Supplementary file 1 — Supplementary Information [file 41467_2022_29880_MOESM1_ESM.pdf]

Supplementary Information

Trends and associated factors for Covid-19 hospitalisation and fatality risk in 2.3 million adults in England

Beaney T[1,2], Neves AL[1], Alboksmaty A[1,2], Ashrafian H[1], Flott K[1], Fowler A[3], Benger JR[4], Aylin P[1,2], Elkin S[5], Darzi A[1], Clarke J[1,6]

1. Patient Safety Translational Research Centre, Institute of Global Health Innovation, Imperial College London, London, SW7 2AZ, United Kingdom
2. Department of Primary Care and Public Health, Imperial College London, London, W6 8RP, United Kingdom
3. NHS England and Improvement, London, SE1 6LH, United Kingdom
4. NHS Digital, 7-8 Wellington Place, Leeds, West Yorkshire, LS1 4AP, United Kingdom
5. National Heart and Lung Institute, Imperial College London, London, SW7 2AZ United Kingdom
6. Centre for Mathematics of Precision Healthcare, Department of Mathematics, Imperial College London, London, SW7 2AZ, United Kingdom

Corresponding Author:
Dr Thomas Beaney
Patient Safety Translational Research Centre, Institute of Global Health Innovation, Imperial College London, London, SW7 2AZ, United Kingdom
Email: thomas.beaney@imperial.ac.uk

Supplementary Figures

Supplementary Fig. 1: Relative probability of hospital admission (panel A) and death (panel B) within 28 days over time in people with Covid-19, compared to the first full week of October 2020, from unadjusted mixed effects logistic regression models (N=2,311,282)

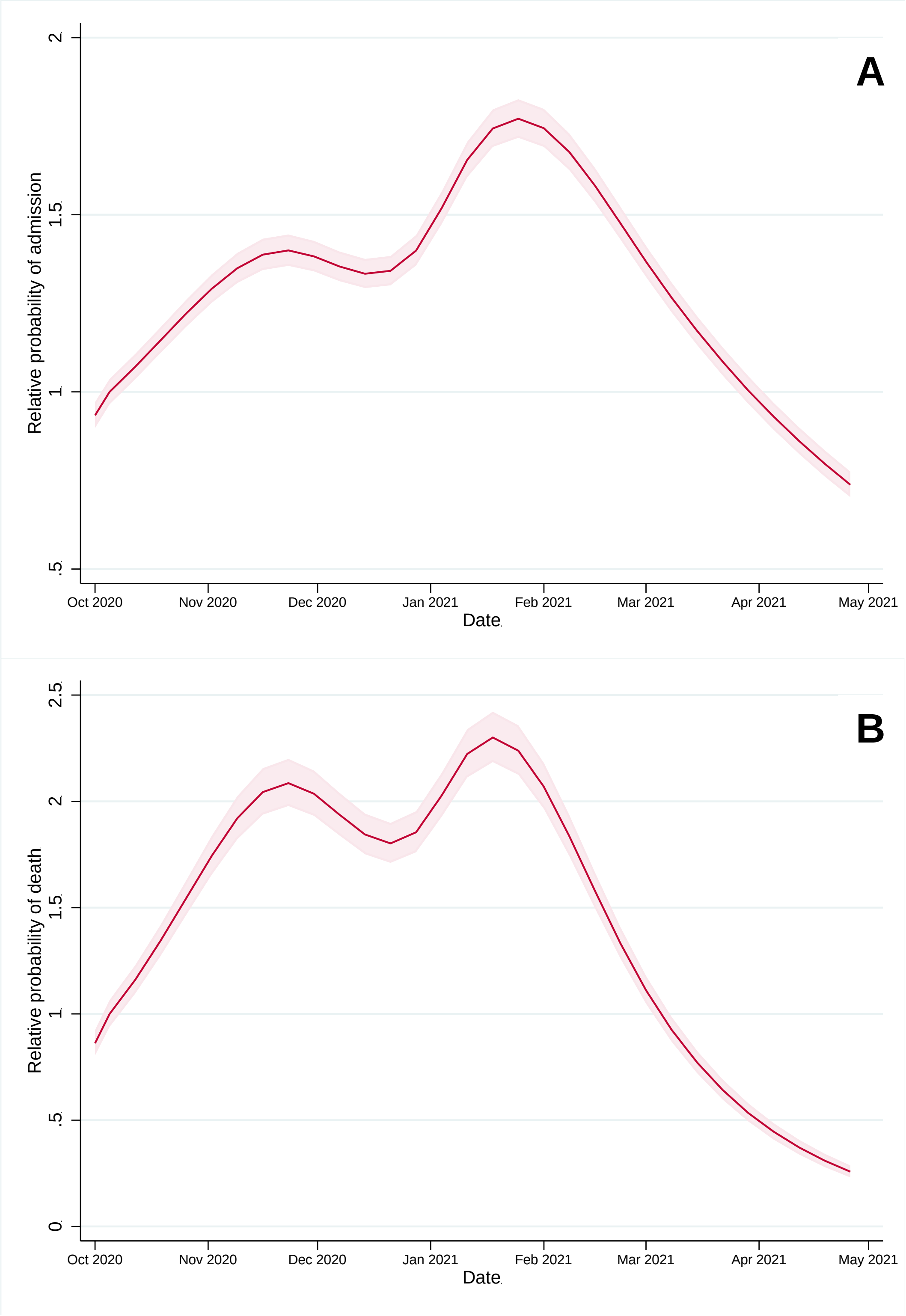

Note: shaded areas represent 95% confidence intervals of the probability estimates.

Supplementary Fig. 2: Case hospitalisation risk (A) and fatality risk (B) over time in people with Covid-19 from unadjusted mixed effects logistic regression models (N=2,311,282)

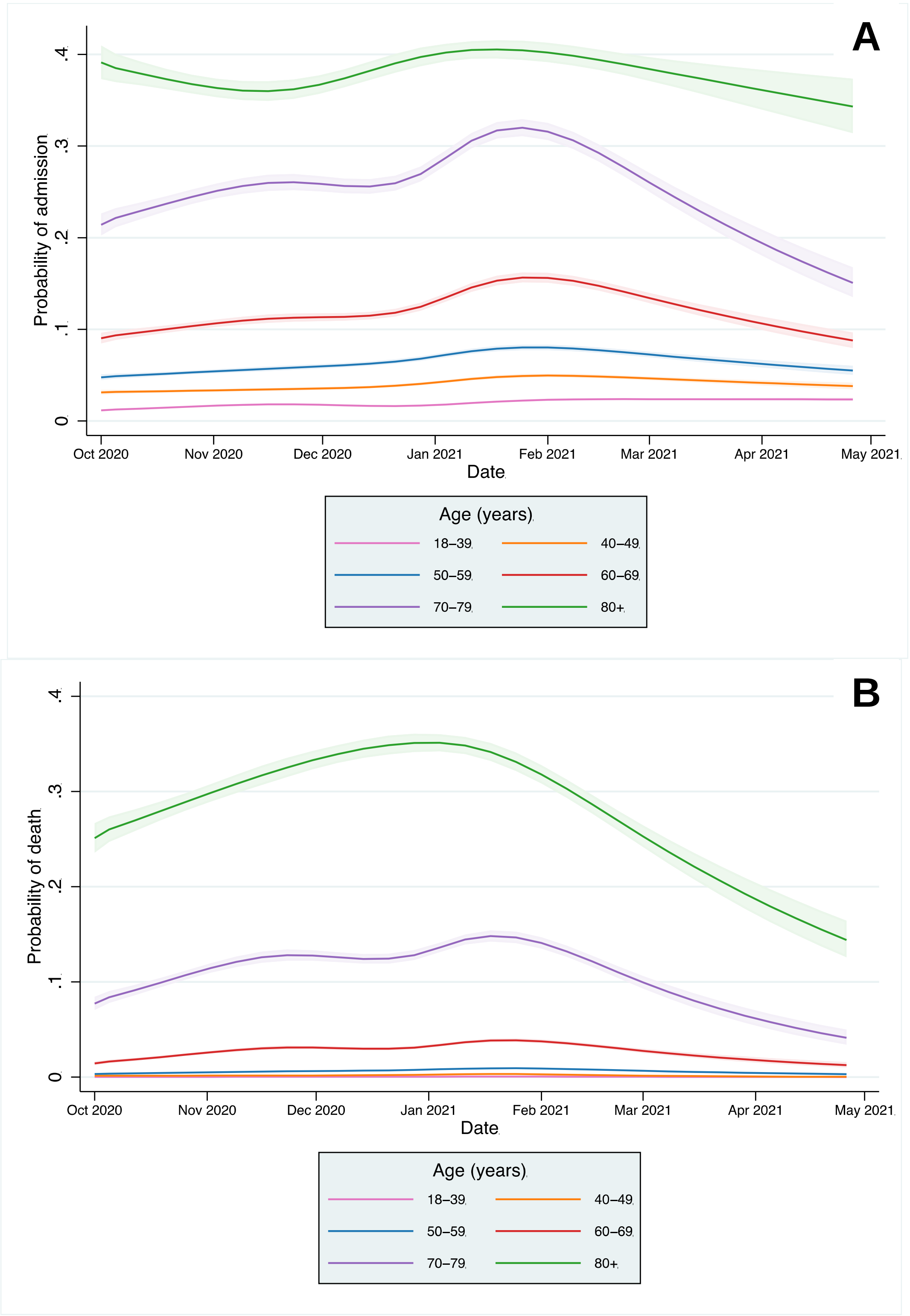

Note: shaded areas represent 95% confidence intervals of the probability estimates.

Supplementary Fig. 3: Calibration plot of predicted vs observed probabilities from mixed effects logistic regression model of 28-day emergency admissions, after multiple imputation (N=2,311,282)

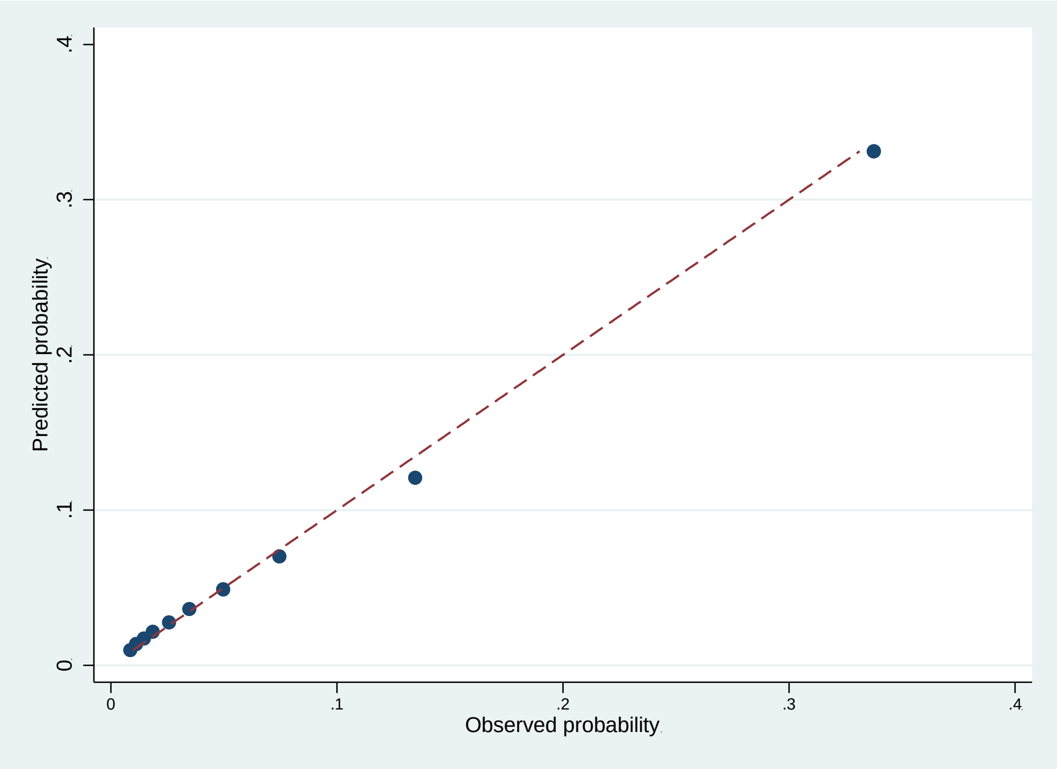

Supplementary Fig. 4: Calibration plot of predicted vs observed probabilities from mixed effects logistic regression model of 28-day mortality, after multiple imputation (N=2,311,282)

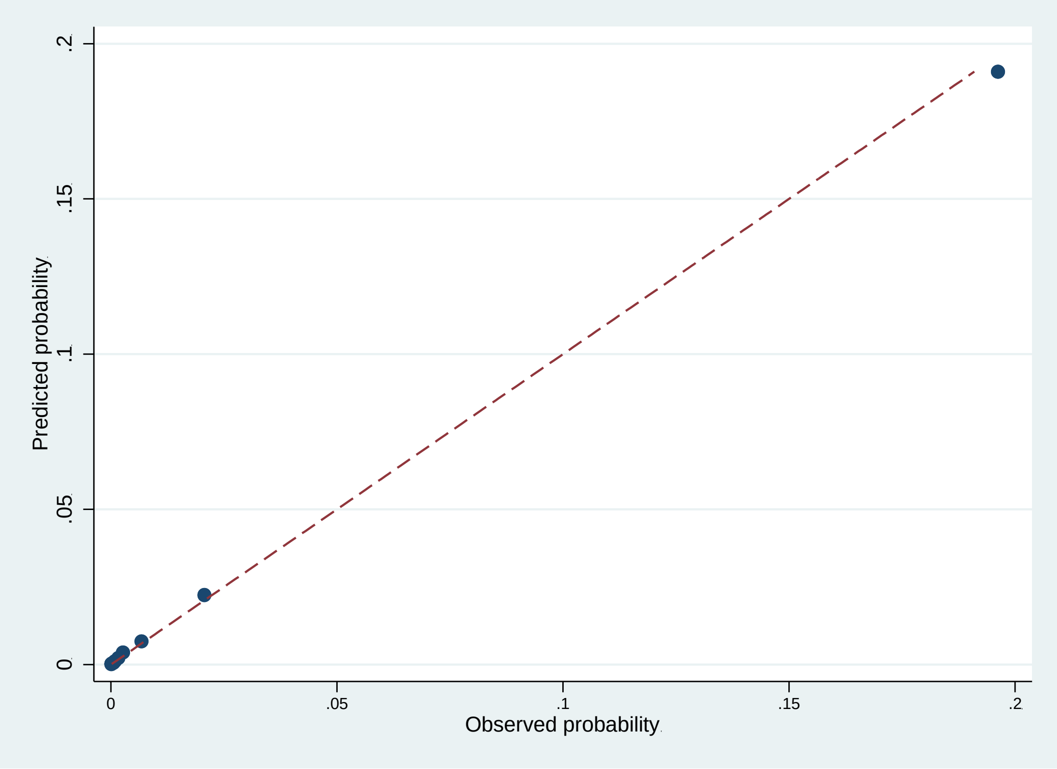

Supplementary Fig. 5: Relative probability of hospital admission (panel A) and death (panel B) within 28 days over time in people with Covid-19, compared to the first full week of October 2020, from fully adjusted mixed effects logistic regression models (N=2,311,282)

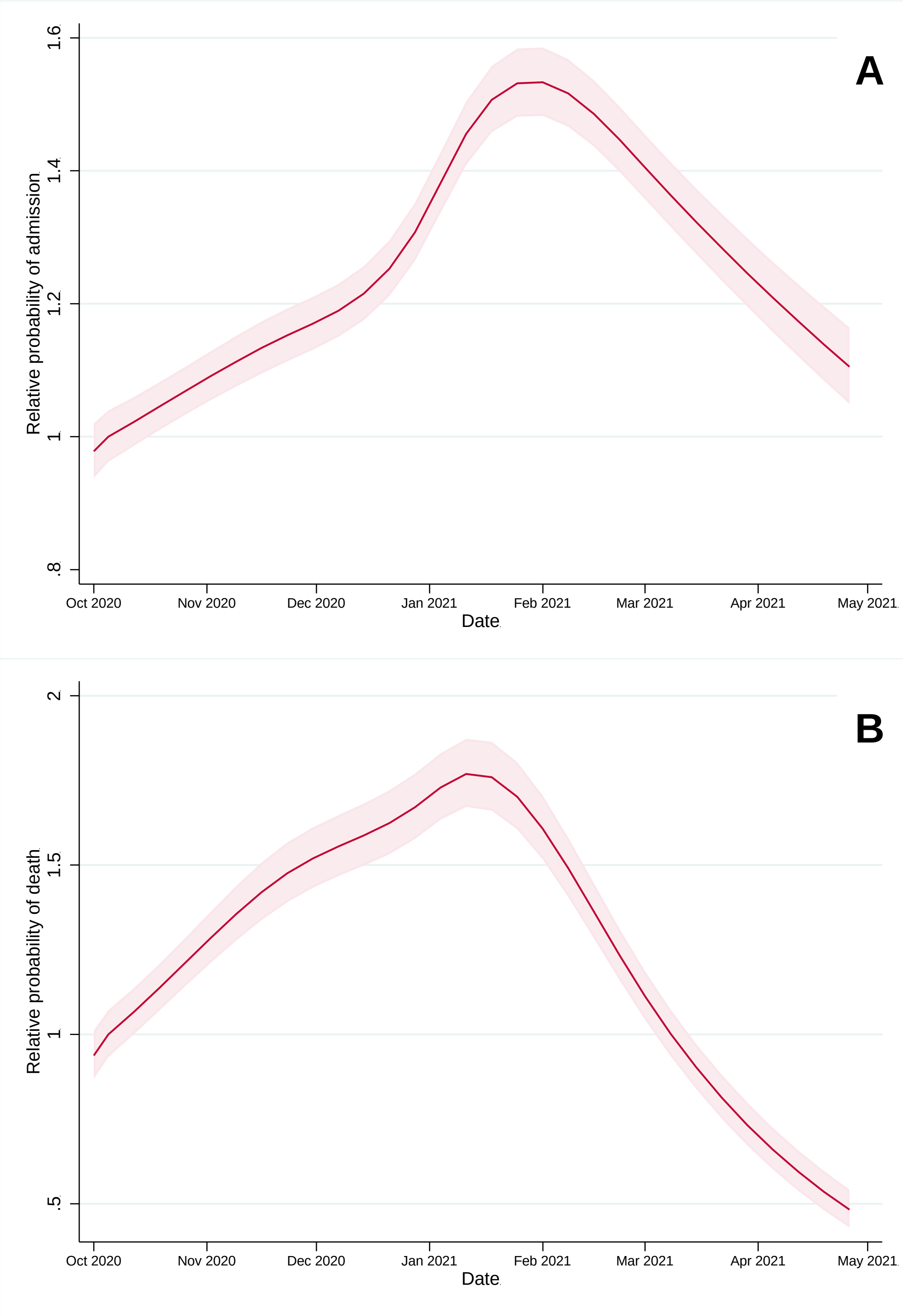

***Full adjustment covariates described in the text. Note this model does not include an interaction between age and time. Shaded areas represent 95% confidence intervals of the probability estimates.**

Supplementary Fig. 6: Relative probability of hospital admission within 28 days over time in people with Covid-19, compared to the first full week of October 2020, from fully adjusted mixed effects logistic regression models (N=2,311,282)

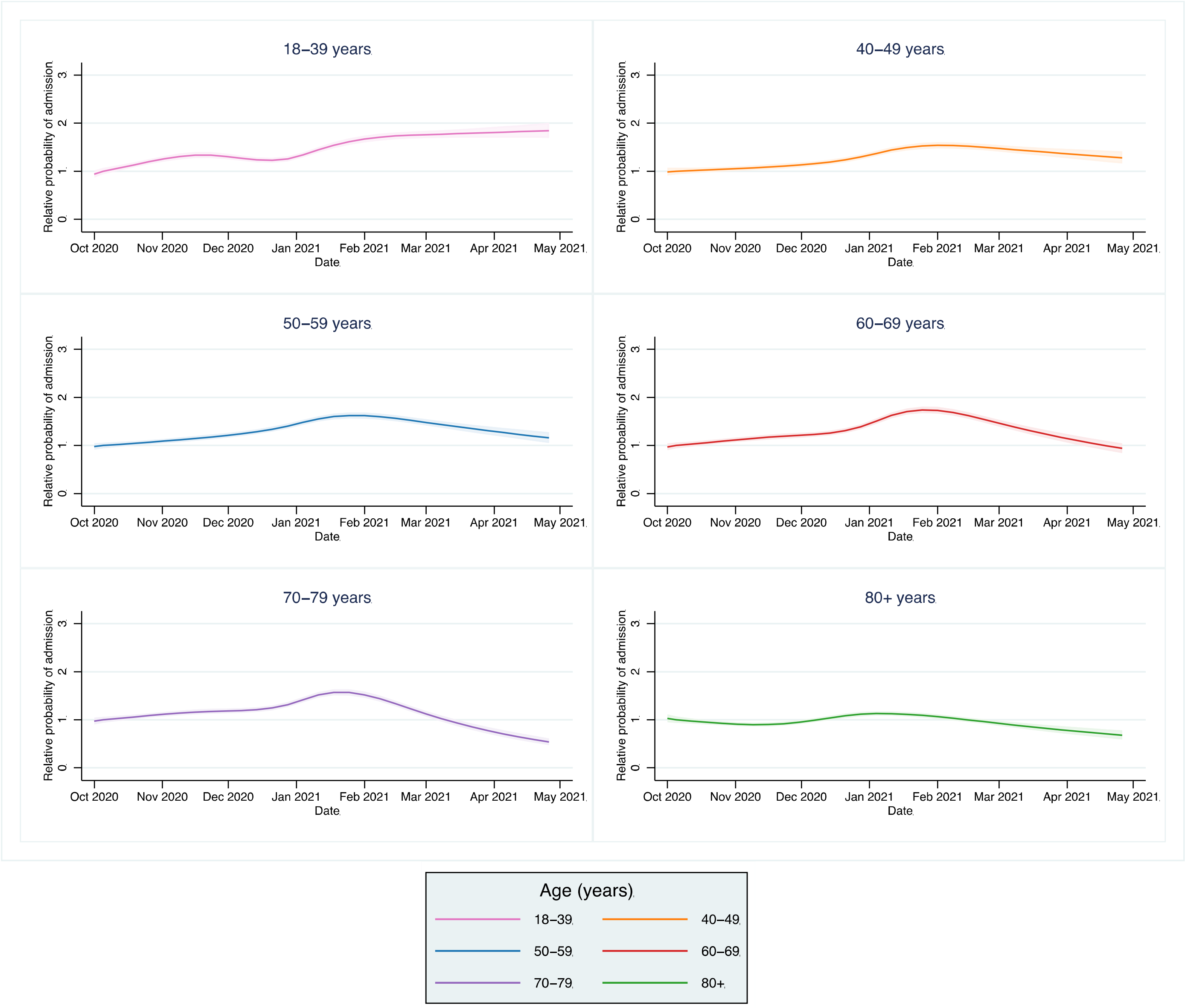

Note: shaded areas represent 95% confidence intervals of the probability estimates

Supplementary Fig. 7: Relative probability of death with 28 days over time in people with Covid-19, compared to the first full week of October 2020, from fully adjusted mixed effects logistic regression models (N=2,311,282)

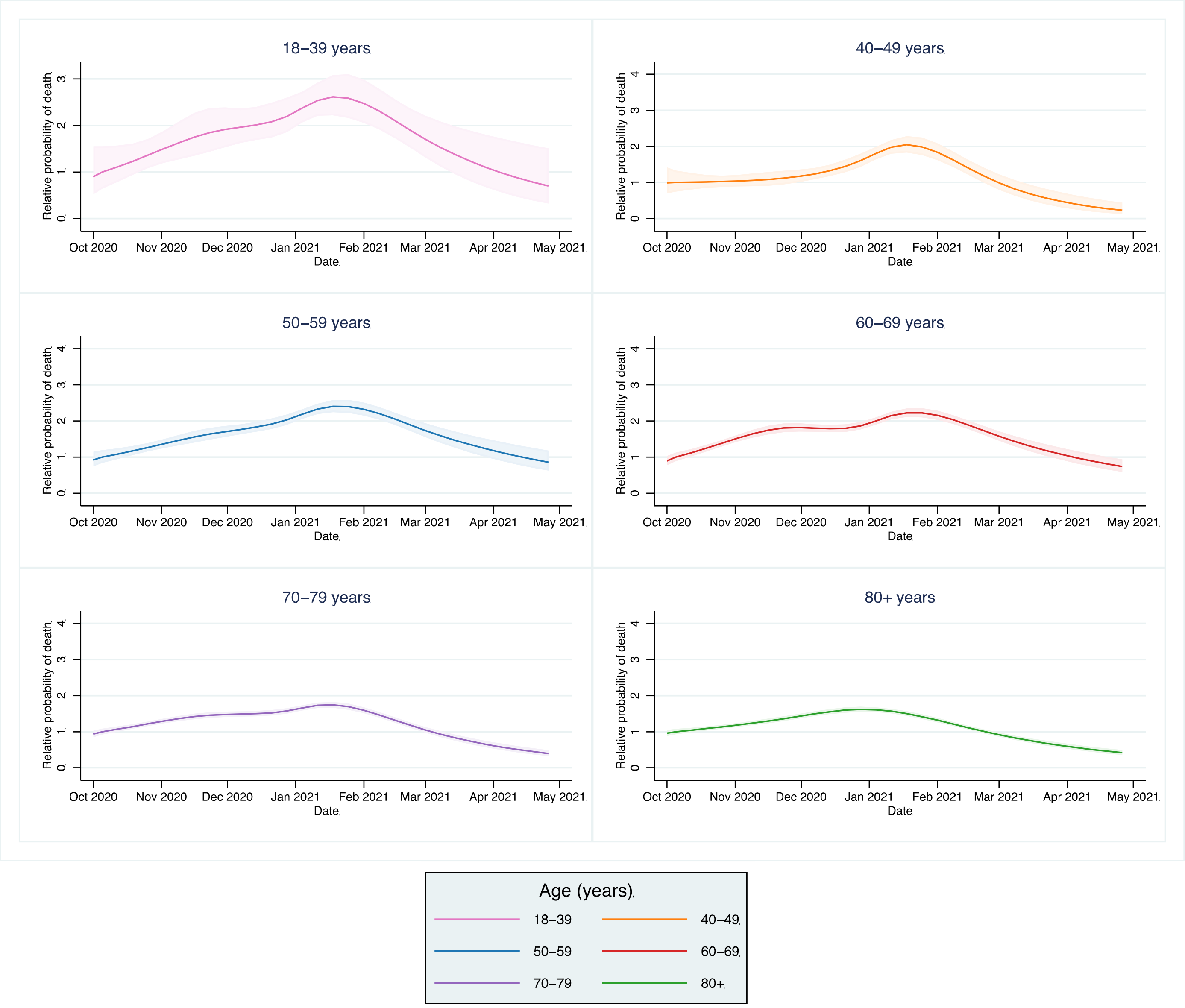

Note: shaded areas represent 95% confidence intervals of the probability estimates

Supplementary Fig. 8: Vaccine uptake at STP level: 60-69 year age group

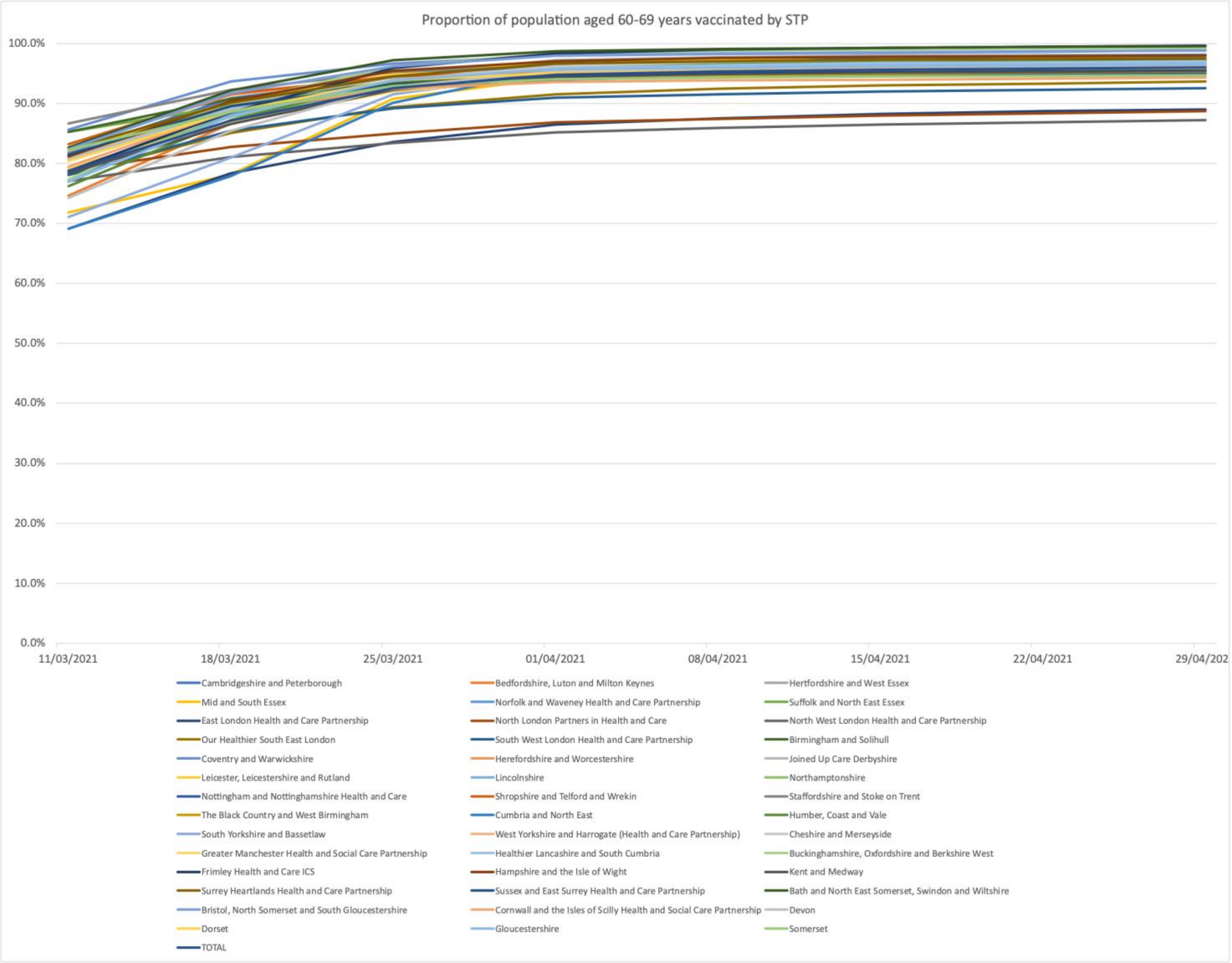

Supplementary Fig. 9: Vaccine uptake at STP level: 70-79 year age group

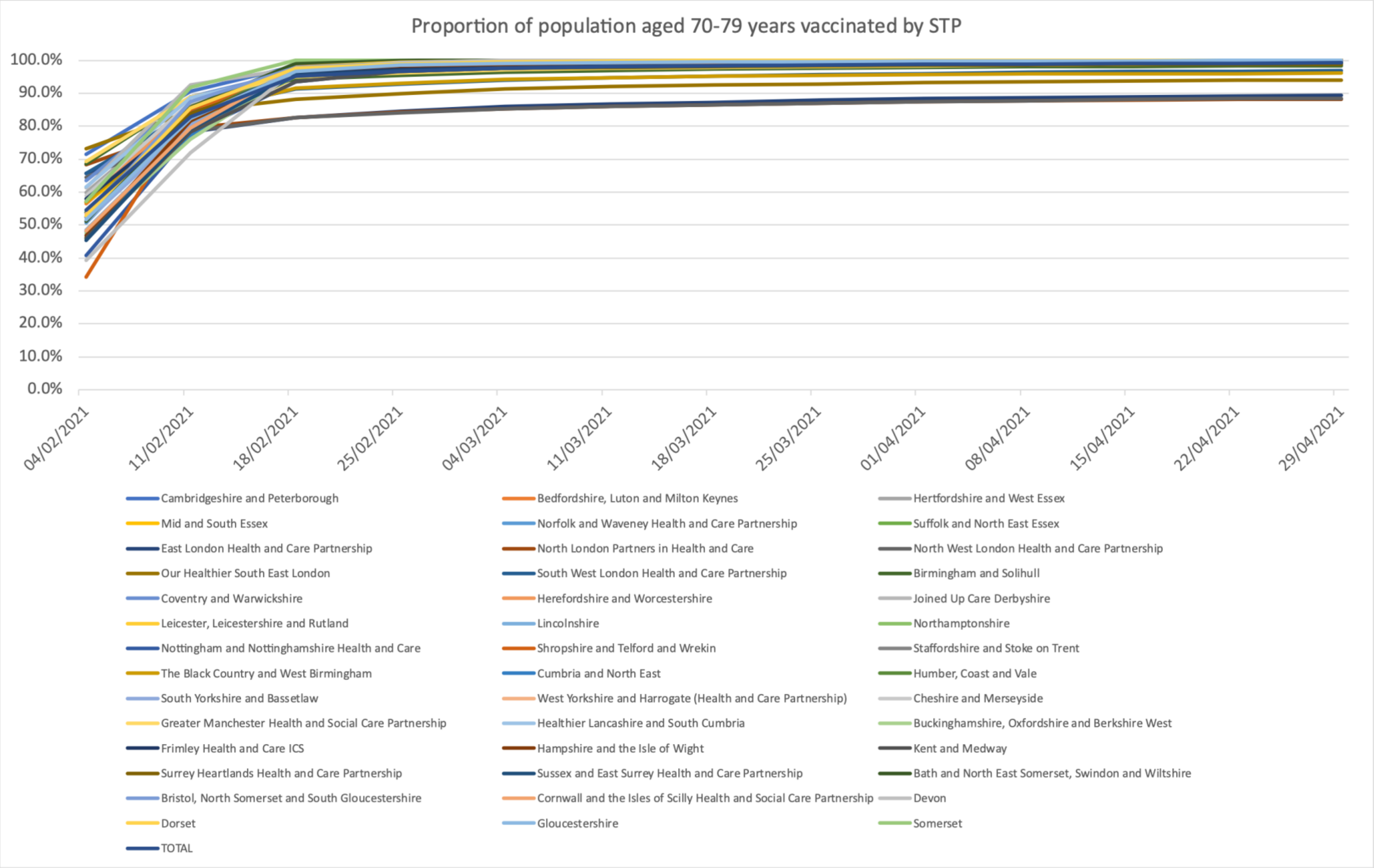

Supplementary Fig. 10: Vaccine uptake at STP level: 80+ year age group

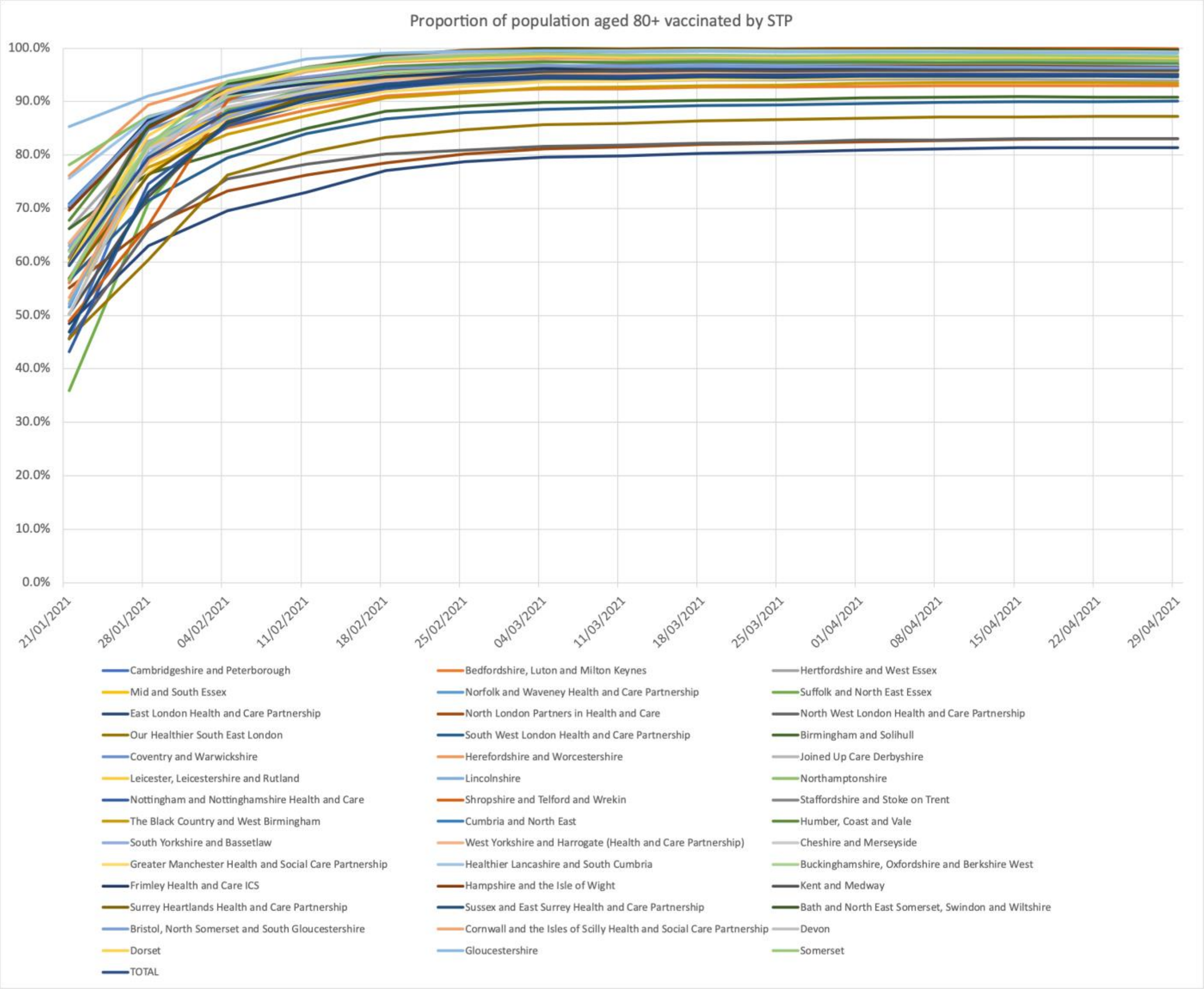

Supplementary Tables

Supplementary Table 1: Percentage of monthly positive Covid-19 tests in each age group from October 2020 to April 2021

Age category (years)	Proportion of monthly positive Covid-19 tests in each age group							
	October	November	December	January	February	March	April	Total
18-39	45.9%	41.1%	43.0%	42.5%	45.1%	49.9%	52.8%	43.6%
40-49	17.4%	19.4%	20.7%	18.5%	18.5%	20.6%	21.2%	19.1%
50-59	18.6%	19.4%	18.7%	19.3%	18.5%	17.0%	14.1%	18.8%
60-69	9.9%	10.4%	9.5%	10.4%	10.0%	7.3%	7.2%	9.9%
70-79	5.1%	5.6%	4.6%	5.1%	4.4%	2.9%	3.0%	4.9%
80+	3.1%	4.2%	3.5%	4.1%	3.5%	2.3%	1.7%	3.7%

Supplementary Table 2: Crude case hospitalisation risk by month of Covid-19 test from October 2020 to April 2021

Age category (years)	Hospital admissions within 28 days of positive test							
	October	November	December	January	February	March	April	Total
**18-39**	1.5%	1.8%	1.8%	2.1%	2.4%	2.6%	2.5%	2.0%
**40-49**	3.4%	3.6%	4.1%	4.8%	5.2%	4.7%	4.2%	4.3%
**50-59**	5.4%	5.7%	6.9%	7.8%	8.2%	7.4%	5.8%	6.9%
**60-69**	10.3%	11.0%	12.8%	14.7%	16.1%	13.4%	8.8%	13.1%
**70-79**	24.0%	25.9%	27.2%	30.9%	31.7%	24.2%	17.7%	28.0%
**80+**	38.1%	37.4%	38.9%	41.5%	39.4%	38.1%	39.2%	39.5%
**All ages**	**5.7%**	**6.7%**	**6.8%**	**8.1%**	**8.0%**	**6.1%**	**4.8%**	**7.1%**

Supplementary Table 3: Crude case fatality risk by month of Covid-19 test from October 2020 to April 2021

Age category (years)	Deaths within 28 days of positive test							
	October	November	December	January	February	March	April	Total
**18-39**	<0.1%	<0.1%	<0.1%	<0.1%	<0.1%	<0.1%	<0.1%	<0.1%
**40-49**	0.2%	0.2%	0.2%	0.3%	0.2%	0.1%	<0.1%	0.2%
**50-59**	0.4%	0.6%	0.7%	0.9%	0.9%	0.5%	0.4%	0.7%
**60-69**	2.1%	2.7%	3.0%	3.6%	3.6%	2.3%	1.7%	3.1%
**70-79**	9.8%	11.6%	13.0%	14.1%	13.3%	8.9%	5.1%	12.5%
**80+**	28.3%	30.7%	34.9%	34.4%	30.3%	22.2%	20.0%	32.4%
**All ages**	**1.7%**	**2.4%**	**2.3%**	**2.8%**	**2.3%**	**1.1%**	**0.7%**	**2.3%**

Supplementary Table 4: Estimated adjusted odds ratios for emergency hospital admission within 28 days for patient-level predictors from multivariable mixed effects logistic regression models (N=2,311,282)

Outcome	Adjusted odds ratio	Standard error	p-value	95% confidence interval	
				Lower	Upper
**Sex**					
Female	Reference				
Male	1.41	0.01	<0.001	1.39	1.42
**Ethnicity**					
White	Reference				
Asian/Asian British	1.36	0.01	<0.001	1.34	1.39
Black/African/Caribbean/Black British	1.40	0.02	<0.001	1.36	1.44
Mixed/Multiple ethnic groups	1.32	0.03	<0.001	1.26	1.39
Other ethnic group	1.65	0.03	<0.001	1.60	1.71
**IMD decile**					
1 (most deprived)	Reference				
2	0.93	0.01	0.54	0.91	0.95
3	0.87	0.01	0.001	0.85	0.89
4	0.84	0.01	0.001	0.82	0.86
5	0.82	0.01	<0.001	0.80	0.84
6	0.77	0.01	<0.001	0.75	0.79
7	0.74	0.01	<0.001	0.72	0.76
8	0.73	0.01	<0.001	0.71	0.75
9	0.71	0.01	<0.001	0.69	0.73
10 (least deprived)	0.68	0.01	<0.001	0.66	0.70
**Body Mass Index**					
Underweight	1.10	0.02	<0.001	1.05	1.14
Healthy weight	Reference				
Overweight	1.24	0.01	<0.001	1.22	1.26
Obese	1.93	0.02	<0.001	1.90	1.97
**Smoking status**					

Never smoker	Reference				
Ex-smoker	1.14	0.01	<0.001	1.13	1.16
Current smoker	0.91	0.01	<0.001	0.90	0.93
**Clinically Extremely Vulnerable**					
No	Reference				
Yes	1.85	0.01	<0.001	1.83	1.88
**Comorbidities**					
Hypertension	1.11	0.01	<0.001	1.10	1.13
Chronic cardiac disease	1.21	0.01	<0.001	1.19	1.23
Chronic kidney disease	1.47	0.03	<0.001	1.41	1.53
Chronic respiratory disease	1.30	0.01	<0.001	1.28	1.32
Dementia	0.94	0.02	0.001	0.90	0.98
Diabetes	1.33	0.01	<0.001	1.31	1.35
Chronic neurological disease (including epilepsy)	1.49	0.02	<0.001	1.46	1.53
Learning disability	2.06	0.06	<0.001	1.94	2.18
Malignancy or immunosuppression	1.18	0.01	<0.001	1.16	1.20
Severe mental illness	1.63	0.03	<0.001	1.58	1.68
Peripheral vascular disease	1.17	0.02	<0.001	1.13	1.22
Stroke or TIA	1.21	0.01	<0.001	1.18	1.24

Models also included age category and time (study week as a restricted cubic spline with five knots) with an age and time interaction (coefficients not shown). Models adjusted for all other predictors displayed in table.

Supplementary Table 5: Estimated adjusted odds ratios for death within 28 days for patient-level predictors from multivariable mixed effects logistic regression models (N=2,311,282)

Outcome	Adjusted odds ratio	Standard error	p-value	95% confidence interval	
				Lower	Upper
**Sex**					
Female	Reference				
Male	1.62	0.02	<0.001	1.58	1.65
**Ethnicity**					
White	Reference				
Asian/Asian British	1.11	0.02	<0.001	1.07	1.16
Black/African/Caribbean/Black British	1.14	0.04	<0.001	1.07	1.22
Mixed/Multiple ethnic groups	1.02	0.06	0.712	0.91	1.15
Other ethnic group	1.07	0.05	0.177	0.97	1.17
**IMD decile**					
1 (most deprived)	Reference				
2	0.94	0.02	0.005	0.90	0.98
3	0.89	0.02	<0.001	0.85	0.93
4	0.86	0.02	<0.001	0.83	0.90
5	0.80	0.02	<0.001	0.77	0.84
6	0.79	0.02	<0.001	0.75	0.82
7	0.75	0.02	<0.001	0.72	0.79
8	0.72	0.02	<0.001	0.69	0.75
9	0.71	0.02	<0.001	0.68	0.75
10 (least deprived)	0.70	0.02	<0.001	0.67	0.74
**Body Mass Index**					
Underweight	1.99	0.06	<0.001	1.87	2.11
Healthy weight	Reference				
Overweight	0.80	0.01	<0.001	0.77	0.82
Obese	1.04	0.02	0.012	1.01	1.07
**Smoking status**					

Never smoker	Reference				
Ex-smoker	1.34	0.02	<0.001	1.31	1.37
Current smoker	1.35	0.02	<0.001	1.31	1.40
**Clinically Extremely Vulnerable**					
No	Reference				
Yes	0.88	0.01	<0.001	0.86	0.90
**Comorbidities**					
Hypertension	1.09	0.01	<0.001	1.07	1.11
Chronic cardiac disease	1.47	0.02	<0.001	1.44	1.50
Chronic kidney disease	2.26	0.06	<0.001	2.15	2.37
Chronic respiratory disease	1.38	0.02	<0.001	1.35	1.41
Dementia	1.67	0.04	<0.001	1.60	1.74
Diabetes	1.44	0.02	<0.001	1.41	1.47
Chronic neurological disease (including epilepsy)	1.55	0.03	<0.001	1.49	1.61
Learning disability	2.46	0.14	<0.001	2.21	2.75
Malignancy or immunosuppression	1.63	0.02	<0.001	1.59	1.67
Severe mental illness	2.29	0.05	<0.001	2.18	2.39
Peripheral vascular disease	1.42	0.03	<0.001	1.35	1.48
Stroke or TIA	1.27	0.02	<0.001	1.24	1.31

Models also include age category and time (study week as a restricted cubic spline with five knots) with an age and time interaction (coefficients not shown). Models adjusted for all other predictors displayed in table.

Supplementary Table 6: Estimated adjusted odds ratios for emergency hospital admission within 28 days for patient-level predictors from multivariable mixed effects logistic regression models for the complete cases (N=1,929,999)

Outcome	Adjusted odds ratio	Standard error	p-value	95% confidence interval	
				Lower	Upper
**Sex**					
Female	Reference				
Male	1.44	0.01	<0.001	1.42	1.46
**Ethnicity**					
White	Reference				
Asian/Asian British	1.37	0.01	<0.001	1.34	1.39
Black/African/Caribbean/Black British	1.40	0.02	<0.001	1.36	1.44
Mixed/Multiple ethnic groups	1.32	0.03	<0.001	1.26	1.38
Other ethnic group	1.67	0.03	<0.001	1.61	1.73
**IMD decile**					
1 (most deprived)	Reference				
2	0.94	0.01	<0.001	0.92	0.96
3	0.88	0.01	<0.001	0.86	0.90
4	0.84	0.01	<0.001	0.82	0.86
5	0.83	0.01	<0.001	0.81	0.85
6	0.78	0.01	<0.001	0.76	0.80
7	0.75	0.01	<0.001	0.73	0.77
8	0.75	0.01	<0.001	0.73	0.77
9	0.72	0.01	<0.001	0.70	0.74
10 (least deprived)	0.70	0.01	<0.001	0.68	0.72
**Body Mass Index**					
Underweight	1.12	0.02	<0.001	1.08	1.17
Healthy weight	Reference				
Overweight	1.25	0.01	<0.001	1.23	1.27
Obese	1.95	0.02	<0.001	1.92	1.98
**Smoking status**					

Never smoker	Reference				
Ex-smoker	1.14	0.01	<0.001	1.12	1.16
Current smoker	0.91	0.01	<0.001	0.90	0.93
**Clinically Extremely Vulnerable**					
No	Reference				
Yes	1.86	0.02	<0.001	1.83	1.89
**Comorbidities**					
Hypertension	1.17	0.01	<0.001	1.15	1.18
Chronic cardiac disease	1.24	0.01	<0.001	1.22	1.26
Chronic kidney disease	1.46	0.03	<0.001	1.40	1.51
Chronic respiratory disease	1.34	0.01	<0.001	1.32	1.35
Dementia	0.98	0.02	0.294	0.94	1.02
Diabetes	1.34	0.01	<0.001	1.32	1.36
Chronic neurological disease (including epilepsy)	1.50	0.02	<0.001	1.46	1.54
Learning disability	2.05	0.06	<0.001	1.94	2.18
Malignancy or immunosuppression	1.20	0.01	<0.001	1.18	1.22
Severe mental illness	1.64	0.03	<0.001	1.59	1.69
Peripheral vascular disease	1.18	0.02	<0.001	1.14	1.23
Stroke or TIA	1.23	0.02	<0.001	1.20	1.26

Models also included age category and time (study week as a restricted cubic spline with five knots) with an age and time interaction (coefficients not shown). Models adjusted for all other predictors displayed in table.

Supplementary Table 7: Estimated adjusted odds ratios for death within 28 days for patient-level predictors from multivariable mixed effects logistic regression models for the complete cases ((N=1,929,999))

Outcome	Adjusted odds ratio	Standard error	p-value	95% confidence interval	
				Lower	Upper
**Sex**					
Female	Reference				
Male	1.62	0.02	<0.001	1.58	1.65
**Ethnicity**					
White	Reference				
Asian/Asian British	1.10	0.02	<0.001	1.06	1.15
Black/African/Caribbean/Black British	1.13	0.04	<0.001	1.06	1.21
Mixed/Multiple ethnic groups	1.02	0.06	0.747	0.91	1.15
Other ethnic group	1.06	0.05	0.235	0.96	1.16
**IMD decile**					
1 (most deprived)	Reference				
2	0.95	0.02	0.021	0.91	0.99
3	0.90	0.02	<0.001	0.86	0.94
4	0.88	0.02	<0.001	0.84	0.92
5	0.81	0.02	<0.001	0.77	0.85
6	0.81	0.02	<0.001	0.77	0.84
7	0.76	0.02	<0.001	0.73	0.80
8	0.75	0.02	<0.001	0.71	0.78
9	0.72	0.02	<0.001	0.69	0.76
10 (least deprived)	0.72	0.02	<0.001	0.69	0.76
**Body Mass Index**					
Underweight	1.98	0.06	<0.001	1.86	2.10
Healthy weight	Reference				
Overweight	0.81	0.01	<0.001	0.78	0.83
Obese	1.05	0.01	0.001	1.02	1.08
**Smoking status**					

Never smoker	Reference				
Ex-smoker	1.34	0.02	<0.001	1.31	1.37
Current smoker	1.35	0.02	<0.001	1.31	1.40
**Clinically Extremely Vulnerable**					
No	Reference				
Yes	0.89	0.01	<0.001	0.87	0.92
**Comorbidities**					
Hypertension	1.18	0.01	<0.001	1.16	1.21
Chronic cardiac disease	1.55	0.02	<0.001	1.52	1.59
Chronic kidney disease	2.25	0.06	<0.001	2.14	2.36
Chronic respiratory disease	1.45	0.02	<0.001	1.42	1.49
Dementia	1.76	0.04	<0.001	1.68	1.84
Diabetes	1.49	0.02	<0.001	1.45	1.52
Chronic neurological disease (including epilepsy)	1.59	0.03	<0.001	1.53	1.66
Learning disability	2.48	0.14	<0.001	2.22	2.77
Malignancy or immunosuppression	1.71	0.02	<0.001	1.67	1.75
Severe mental illness	2.35	0.06	<0.001	2.25	2.46
Peripheral vascular disease	1.42	0.03	<0.001	1.36	1.49
Stroke or TIA	1.30	0.02	<0.001	1.26	1.34

Models also include age category and time (study week as a restricted cubic spline with five knots) with an age and time interaction (coefficients not shown). Models adjusted for all other predictors displayed in table.

Supplementary Methods

Data cleaning

This section describes the cleaning process for each dataset.

Covid-19 testing data

Testing data was provided through the Public Health England Second Generation Surveillance System (SGSS). This dataset captures routine laboratory data on infectious diseases for England, including Covid-19, with all diagnostic laboratories required to notify positive test results within 24 hours.[1] Data included 3,251,225 tests performed from 1st October 2020 to 30th June 2021, inclusive.

Provided data included test date and result date. 99% of results were reported within 5 days of the test, and 6,544 (0.2%) were reported more than 7 days from date of test. Where the result data occurred before the test date. In 1,603 cases, the result date was recorded as prior to the test date. In these instances, where the difference between the testing date and reporting date was 7 days or less, the test date and reporting date were swapped. In the 191 instances where reporting date was more than 7 days before the testing date, the test was excluded.

For this analysis, only tests performed up to 30th April 2021 were included (after swapping test and result dates where applicable), given that secondary care data was available up until the end of May 2021. Of the 2,925,356 positive Covid-19 tests, 2,349,237 (80.3%) were from Pillar 2 testing, 561,570 (19.2%) from Pillar 1, and 14,549 (0.5%) from Pillar 4.[2] Test type was recorded for Pillar 2 tests only, and of these, 2,248,497 (95.7%) were Polymerase Chain Reaction (PCR) tests, 100,740 (4.3%) were lateral flow tests.

In these analyses, only the first case of a positive test where more than one test was recorded for any individual (2,533,504). Any patient resident in a Clinical Commissioning Group (CCG) outside of England, or in NHS commission hub CCGs, were excluded resulting in testing population of 2,433,768 individuals.

Shielded Patient List

Identification of high-risk patients, designated 'Clinically Extremely Vulnerable' (CEV) was provided via a linkage from the NHS Digital Shielded Patient List (SPL) to the primary care record.[3] Patients with any of the conditions listed on p4 below were designated as high risk.[4] Code lists for the conditions are available to download from NHS Digital.[5] In addition, from February 2021, people were also added to the list if identified as high risk using the QCovid risk prediction model, which combines factors including age, sex, ethnicity, BMI as well as specific conditions.[6] Thresholds of mortality risk for inclusion in the SPL were an absolute risk of 0.5% or higher, or a relative risk of 10 times the baseline risk of a person with the same age and sex.[4] GP practices and NHS Trusts were also able to add and remove patients from the SPL based on clinical judgment and individual risk assessments.[7]

NHS Digital criteria for Clinically Extremely Vulnerable patients (source: https://digital.nhs.uk/coronavirus/shielded-patient-list/risk-criteria):

- solid organ transplant recipients
- people with severe respiratory conditions including all cystic fibrosis, severe asthma and severe chronic obstructive pulmonary (COPD)
- people with rare diseases and inborn errors of metabolism that significantly increase the risk of infections (such as Severe combined immunodeficiency (SCID), homozygous sickle cell)
- people on immunosuppression therapies sufficient to significantly increase risk of infection
- people who have problems with their spleen, for example have had a splenectomy
- adults with Down’s syndrome
- adults on dialysis with kidney impairment (Stage 5 Chronic Kidney Disease)
- women who are pregnant with significant heart disease, congenital or acquired
- people with cancer who are undergoing active chemotherapy
- people with lung cancer who are undergoing radical radiotherapy
- people with cancers of the blood or bone marrow such as leukaemia, lymphoma or myeloma who are at any stage of treatment
- people having immunotherapy or other continuing antibody treatments for cancer
- people having other targeted cancer treatments which can affect the immune system, such as protein kinase inhibitors or PARP inhibitors
- people who have had bone marrow or stem cell transplants in the last 6 months, or who are still taking immunosuppression drugs

Primary care data

Primary care data came from the General Practice Extraction Service (GPES) Data for Pandemic Planning and Research (GDPPR).[8] Data included month and year of birth, sex, ethnicity, Lower Layer Super Output Area (LSOA) of residence, a marker for CEV status (linked from SPL), and a marker for residence in a care home. LSOA was used to link to 2019 deciles of Index of Multiple Deprivation (IMD).[9] Age was calculated from date of positive Covid-19 test, assuming a birthdate on the 15th day of the month.

Entries include a date to which each journal item applies, and a date on which the journal item was recorded. The former was used in priority, but where missing, was replaced with the journal item recording date. For LSOA, CEV status and care home residence, only entries occurring up to the date of positive Covid-19 test were included. For month and year of birth, sex and ethnicity, if no entry were included prior to the date of Covid-19 test, then the earliest recorded entry after the test was included.

Secondary care data

Data on hospital admissions came from the Hospital Episode Statistics (HES) data set up to 31st May 2021, linked to Office for National Statistics (ONS) data on death registrations up to 5th July 2021.[10] Entries were excluded where missing admission dates, provider Trust code, or patient deidentified ID. Where age was missing from GDPPR, it was derived from month and year of birth in HES using the same approach as for GDPPR.

Where multiple admission episodes were recorded within a spell, a single spell start and end date were created. Non-emergency hospital admissions were excluded from analyses. A binary indicator was created for any admission within 28 days of positive Covid-19 test. A second indicator was created for death (of any cause) within 28 days of positive Covid-19 test.

Co-morbidities

SNOMED codes were included in the GDPPR dataset pertaining to specific SNOMED code cluster reference sets provided by NHS Digital.[8] 6,485 unique codes were identified from

GDPPR. Codes were reviewed manually by authors TB and JC and removed if not relevant or assigned to the minimal number of relevant code clusters.

SNOMED reference clusters were aggregated into hierarchies of similar conditions. Codes in each higher-order cluster were then reviewed to ensure groupings of relevant codes and twelve relevant chronic disease categories were selected: hypertension, chronic cardiac disease, chronic kidney disease, chronic respiratory disease, dementia, diabetes, chronic neurological disease (including epilepsy), learning disability, malignancy/immunosuppression, severe mental illness, peripheral vascular disease and stroke/transient ischaemic attack (TIA). Categories for chronic respiratory disease, diabetes, epilepsy, malignancy/immunosuppression and severe mental illness included relevant medication codes. Broad diagnostic categories of diagnoses were chosen, as certain medications were not diagnostic of more granular diagnostic categories (for example, use of a long-acting bronchodilator/inhaled corticosteroid in both COPD and asthma).

For each patient in GDPPR, all relevant diagnostic codes prior to the study index date (date of positive Covid-19 test) were considered diagnostic. In cases where the latest SNOMED code indicated resolution of a condition (eg ‘Atrial fibrillation resolved (finding)’), then the diagnosis was excluded for that patient. SNOMED codes relating to drug codes were only included up to 2 years prior to the index date.

A full list of codes within each diagnostic category are available in our GitHub repository: https://github.com/tbeaney/Imperial-COh-evaluation

BMI categorisation

SNOMED codes for BMI were either diagnostic categories (eg ‘Body mass index 30+ - obesity (finding)’ or value codes (eg. ‘Body mass index (observable entity)’). Values were extracted and BMI was categorised according to the standard World Health Organisation classification of underweight (<18.5 kg/m^2), healthy weight (18.5-24.9 kg/m^2), overweight (25.0-29.9 kg/m^2) and obese (≥30.0 kg/m^2). Value codes outside of the range 5.0-100.0 kg/m2 were excluded. SNOMED codes which spanned more than one category (eg ‘Increased body mass index (finding)’) and child BMI categories were also excluded.

Smoking categorisation

Smoking status was categorised into 'never-smoker', 'ex-smoker' and 'current smoker' according to the latest SNOMED code prior to and including the index date. For any patient where the latest SNOMED code indicated 'never-smoker', but a prior record indicated active smoking, then the patient was re-categorised as 'ex-smoker.

Statistical analysis

This section provides further detail on the statistical analysis approach. Some of the text here is repeated from the manuscript for clarity.

Statistical analysis

Mixed effects logistic regression was used to model the association between each outcome with age and time (model 1) and age and time, along with patient level covariates (model 2), using the *melogit* command in Stata. Clinical Commissioning Group of residence (according to the date of test) was included as a random intercept into all models.

Time was modelled as a restricted cubic spline, representing the week of the Covid-19 test (with week commencing on a Monday) to allow for flexibility in modelling the relationship with the outcomes. A range of number of knots from three to six were considered, with a five-knot spline chosen for analyses based on the Akaike Information Criteria, with knots placed at equally spaced percentiles.[1] Interaction terms were included between time splines and age category, to allow for varying time trends in different age groups. For plots of the CFR and CHR at the whole population level, age was removed from model 1 (Figure S3) and the age-time interaction was removed from model 2 (Figure S5).

Multiple imputation

Multiple imputation using chained equations was used to impute missing values in patient-level covariates sex, ethnicity, IMD decile, BMI category and smoking status. Age was complete in the data set, according to the exclusion criteria. Co-morbidities and Clinically Extremely Vulnerable (CEV) status were assumed to be absent if not recorded, and so by definition included no missing data. Missingness in covariates was assumed to be missing at

random, i.e. dependent on the remaining observed data. The *mi impute chained* command in Stata was used, with a logit model for sex and a multinomial logit model for ethnicity, IMD decile, BMI category and smoking status. All variables in the analysis models were also included in the imputation model: age, time splines, age by time spline interactions, CEV status and co-morbidities.[2]

A burn in of 10 iterations was deemed to be sufficient for convergence. Convergence was assessed using a trace plot with 25 iterations, and there was no indication of any trend in the mean or standard deviation of the imputed covariates. In total, 15 imputation sets were created, corresponding roughly to the percentage of missing data in the covariate with most missing values (BMI with 14.0% missing values). In analysis models, the Monte Carlo error of the estimates was consistently less than 10% of the standard errors, indicating satisfactory precision and no requirement for further imputations. Estimates from each imputed data set were combined using Rubin's rules, applying the Stata *mi estimate* command.

Predicted probabilities

For each outcome, the predicted probability of the outcome was calculated within each age group and study week stratum to give age- and time- specific Case Hospitalisation Risk (CHR) and Case Fatality Risk (CFR). These were calculated using the fixed portion of the model (assuming zero random effects). Although using only fixed model components can lead to mis-calibration compared to marginal predictions, simulation studies have shown this to be minimal where the where the intra-class correlation (ICC) is less than 0.15.[3] The ICC in fully adjusted mixed effects logistic regression models for admission and death were 0.0078 and 0.0087, respectively, suggesting minimal residual variation explained by clustering at CCG level.

Predicted probabilities were calculated for each of the outcome models, and observed probabilities calculated for each decile of predicted probability. Calibration plots of deciles of predicted against observed probabilities were plotted. Figures S1 and S2 show the plots for admissions and mortality, respectively in the fully adjusted model (model 2) after imputation. These indicated a linear association between predicted and observed probability and no evidence of mis-calibration of the model.

Analyses were conducted in the Big Data and Analytics Unit Secure Environment, Imperial College. Python v3.9.5 and Pandas v1.2.3 were used in data management. Regression models were conducted in Stata v17.0.

Vaccination uptake

Weekly vaccine uptake rates were plotted at Sustainability and Transformation Partnership (STP) level for each age group from the date of earliest data availability until study end date using publicly available data from NHS England.[4] Plots were constructed using Microsoft Excel.

Supplementary References:

1. Public Health England. Laboratory reporting to Public Health England: A guide for diagnostic laboratories [Internet]. 2020 [cited 2021 Sep 30];Available from: https://assets.publishing.service.gov.uk/government/uploads/system/uploads/attachment_data/file/926838/PHE_Laboratory_reporting_guidelines_October-2020-v3.pdf
2. Department of Health & Social Care. COVID-19 testing data: methodology note [Internet]. 2021 [cited 2021 Oct 6];Available from: https://www.gov.uk/government/publications/coronavirus-covid-19-testing-data-methodology/covid-19-testing-data-methodology-note
3. NHS Digital. Shielded Patient List [Internet]. [cited 2022 Feb 26];Available from: https://digital.nhs.uk/coronavirus/shielded-patient-list
4. NHS Digital. Shielded Patient List Risk Criteria [Internet]. 2021 [cited 2021 Sep 22];Available from: https://digital.nhs.uk/coronavirus/shielded-patient-list/risk-criteria
5. NHS Digital. Shielded Patient List: Annexes [Internet]. [cited 2022 Feb 26];Available from: https://digital.nhs.uk/coronavirus/shielded-patient-list/methodology/annexes
6. NHS Digital. Shielded Patient List: COVID-19 Population Risk Assessment [Internet]. [cited 2022 Feb 26];Available from: https://digital.nhs.uk/coronavirus/risk-assessment/population
7. NHS Digital. Guidance for clinicians about the COVID-19 Clinical Risk Assessment Tool [Internet]. [cited 2022 Feb 26];Available from: https://digital.nhs.uk/coronavirus/risk-assessment/clinical-tool/guidance-for-clinicians
8. NHS Digital. General Practice Extraction Service (GPES) Data for pandemic planning and research: a guide for analysts and users of the data [Internet]. 2021 [cited 2021 Sep 30];Available from: https://digital.nhs.uk/coronavirus/gpes-data-for-pandemic-planning-and-research/guide-for-analysts-and-users-of-the-data
9. Ministry of Housing, Communities & Local Government. English indices of deprivation 2019 [Internet]. [cited 2020 Oct 18];Available from: https://www.gov.uk/government/statistics/english-indices-of-deprivation-2019
10. Digital N. Hospital Episode Statistics (HES) [Internet]. [cited 2021 Oct 6];Available from: https://digital.nhs.uk/data-and-information/data-tools-and-services/data-services/hospital-episode-statistics
